# Rectosigmoid Intussusception Due to a Nonmalignant Etiology: An Extremely Rare Cause of Acute Lower Gastrointestinal Bleeding in an Elderly Patient

**DOI:** 10.14309/crj.0000000000001706

**Published:** 2025-05-16

**Authors:** Kashif Ali, Samra Sohail, Grecia Dominguez Rivera, Valeska Balderas

**Affiliations:** 1 Department of Internal Medicine, University of Texas Rio Grande Valley, TX; 2 Department of Internal Medicine, Dow University of Health Sciences, Karachi, Pakistan; 3 Division of Gastroenterology and Nutrition, University of Texas Health San Antonio, TX; 4 Department of Internal Medicine, Division of Gastroenterology, University of Texas Rio Grande Valley, TX

**Keywords:** intussusception, lower gastrointestinal bleeding, elderly patient

## Abstract

An intussusception at the rectosigmoid junction with acute gastrointestinal bleeding is exceedingly rare. We present a case of an 88-year-old man who presented in the emergency department with bilateral lower quadrant abdominal pain and frank rectal bleeding for 2 days. A computed tomography abdomen pelvis angiogram with contrast showed a target sign, and a flexible sigmoidoscopy showed congested bowel, ischemia, frank blood, and intussusception at the rectosigmoid junction. He underwent an exploratory laparotomy with the removal of the affected part. The pathology and the peritoneal fluid cytology results came out negative for dysplasia and malignancy.

## INTRODUCTION

Intussusception is rarely found in the elderly population. While it is 20 times more common in the pediatric population, adult cases account for less than 5% of cases and around 1%–3% of intestinal obstruction cases in this age group.^1–3^

Early diagnosis is crucial due to the risk of significant complications, including bowel obstruction, ischemia, and necrosis. However, its nonspecific clinical presentation often makes early diagnosis challenging. Intussusception at the rectosigmoid junction presenting with acute gastrointestinal (GI) bleeding is exceedingly rare, with no previous cases reported in the literature. We present a case of rectosigmoid intussusception, an unusual cause of acute lower GI bleeding in an elderly patient.

## CASE REPORT

**Figure 1.**
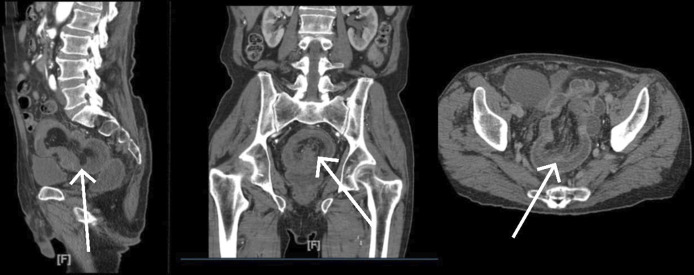
Multiple computed tomography scan views showing a distended rectosigmoid and rectum with target sign at the rectosigmoid junction consistent with intussusception.

An 88-year-old Hispanic man with a medical history of asthma, hypertension, hyperlipidemia, and home hospice (revoked before the visit) for advanced chronic obstructive pulmonary disease on 5 L home oxygen was brought to the Emergency Department due to bilateral lower quadrant abdominal pain and frank bleed per rectum for the last 2 days. He denied any recent hematemesis, fever, chills, diarrhea, chest pain, or hematuria. Vital signs showed tachypnea with a respiratory rate of 28, blood pressure of 108/44, and oxygen saturation 93% on 5 L nasal cannula. Abdominal examination revealed a soft, nondistended abdomen with suprapubic tenderness and frank bleeding per rectal examination. Physical examination was otherwise unremarkable. The initial laboratory investigations showed anemia with hemoglobin of 10.2 g/dL, hematocrit of 34.1%, leukocytosis with a white cell count of 17 × 10^3^/μL, and platelet count 233 × 10^3^/μL. The coagulation profile was within the normal range. The following day, hemoglobin dropped from 10.1 to 8.0, and he was transfused with 1 unit of packed red blood cells. A computed tomography (CT) abdomen pelvis angiogram with contrast using 100 cc omnipaque 350 injection was performed which showed a distended rectosigmoid and rectum with Target sign at the rectosigmoid junction consistent with intussusception. There was no proximal dilation noted (Figure 1).

**Figure 2.**
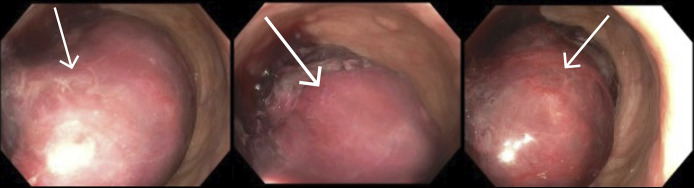
Flexible sigmoidoscopy showing congested bowel, ischemia, frank blood, and intussusception at the rectosigmoid junction.

**Figure 3.**
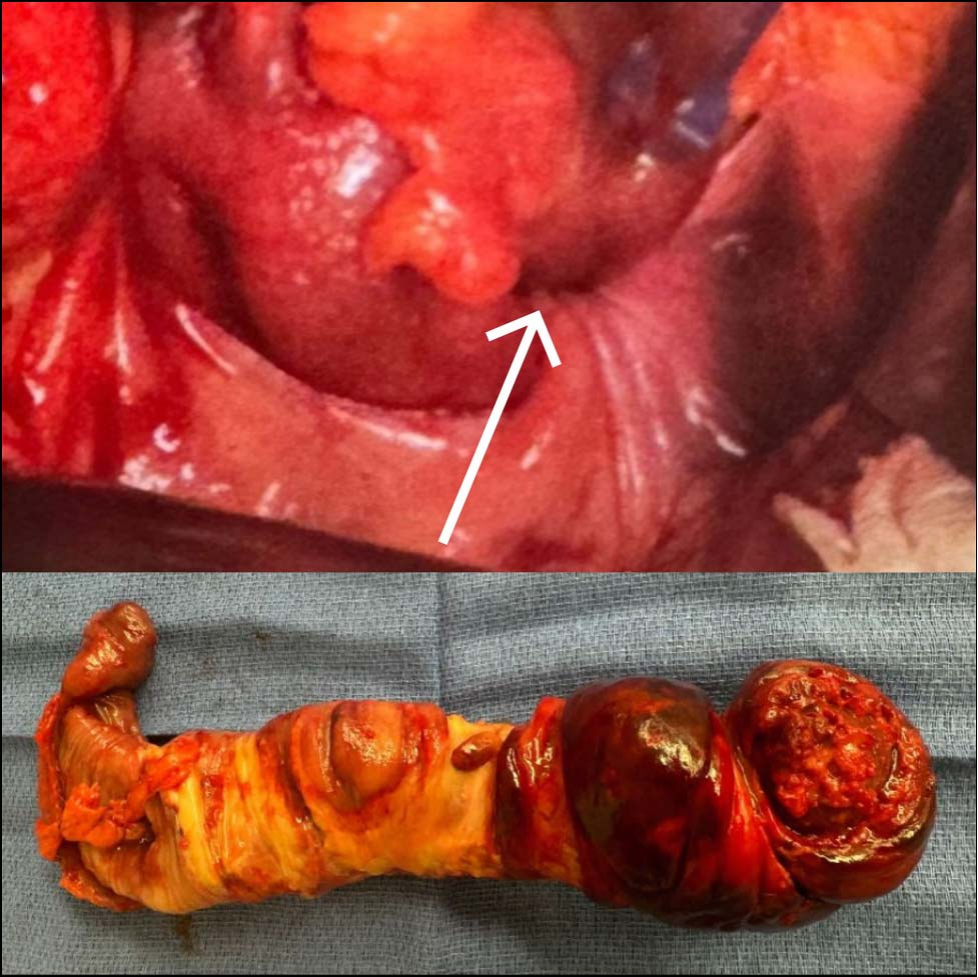
Resected specimen at rectosigmoid junction.

**Figure 4.**
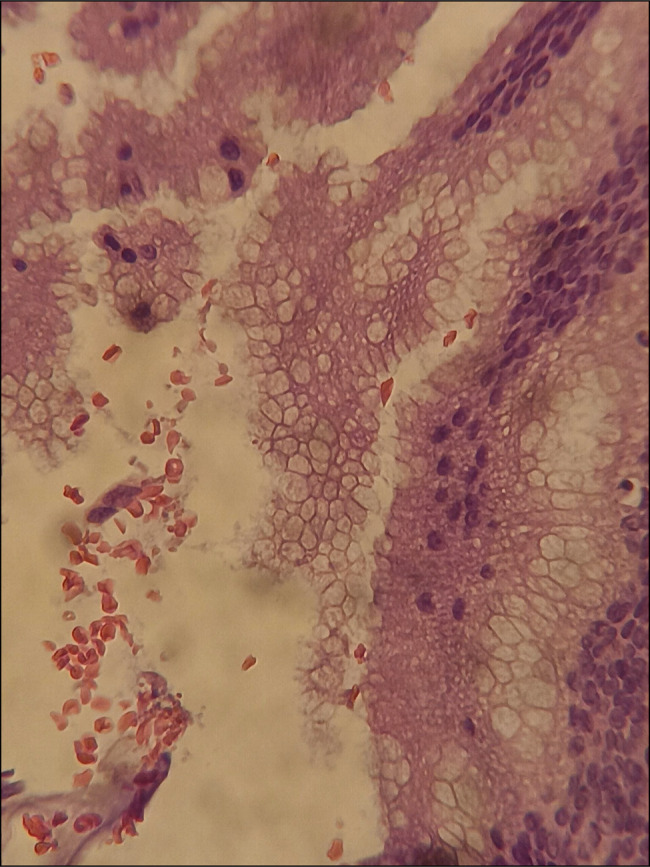
Histology shows benign mucinous epithelial cells (40×).

He underwent a flexible sigmoidoscopy that revealed congested bowel, ischemia, frank blood, and intussusception at the rectosigmoid junction (Figure 2). The patient had no documented history of prior colonoscopies, and therefore, no previous imaging was available for comparison. General surgery was consulted, and an exploratory laparotomy was performed. He was found to have rectosigmoid intussusception, and the affected rectosigmoid section was resected (Figure 3). The pathology of the resected segment showed multifocal patterns of acute ischemia with edema, congestion, mural, and was negative for granulomas, neoplasia, and malignancy (Figure 4). Peritoneal fluid cytology was performed following peritoneal wash, and it was also negative for malignancy. Postoperative care was uncomplicated.

Two weeks later, the patient presented with erythema and mucopurulent discharge from the surgical site, consistent with surgical site infection which was managed nonoperatively with intravenous antibiotics and wound care, and the patient was discharged back.

## DISCUSSION

Intussusception can be classified as primary (idiopathic) when no lead point is identified and secondary with an underlying lead point. In adults, most cases are secondary, with only about 10% idiopathic, unlike the pediatric population where 90% are idiopathic.^1,3–5^ The lead point can be further classified as malignant or benign. In the small intestine, most lead points are benign, including benign neoplasms, Meckel's diverticulum, appendix, adhesions, and inflammatory lesions with malignancy accounting for only 25%–30% of cases. Conversely, in the large bowel, 60%–65% of intussusception cases are associated with malignancy.^2,4^ No lead point was identified in our case, and malignancy was ruled out due to negative findings of malignancy on tissue biopsy or peritoneal fluid cytology.

Diagnosing patients can be challenging as they present with nonspecific symptoms, ranging from acute abdominal pain and nausea to chronic, intermittent symptoms that may be confused with other conditions that present similarly such as appendicitis, ovarian torsion, abdominal hernias, volvulus, and Meckel diverticulum.^1,6^ Adults rarely present with the typical triad of abdominal pain, bloody stools, and palpable abdominal mass.^4,5,7^ Our patient presented with acute pain and bleeding but no palpable mass on examination.

Early diagnosis is crucial to ensure timely intervention thus preventing serious complications. Diagnostic imaging plays an essential role, with CT as the gold standard due to its high sensitivity and accuracy, reported to range from 58% to 100%.^2–5^ It allows for the identification of lead point, the location of the bowel involved, and the recognition of specific signs like the “target” and “sausage-shape” appearance.^1,3^ Flexible lower GI endoscopy may also aid in the diagnosis and treatment of subacute and chronic presentations.

Management should be individualized based on the location, etiology, and clinical presentation of the patient. Reduction before surgery has been debated, as it helps preserve bowel length. For small-bowel intussusception, where most cases are benign, reduction is generally recommended if malignancy is not suspected, and the bowel is viable. However, for colonic intussusception, where the risk of malignancy is significantly higher, surgical resection is the preferred approach.^3^ In acute cases where underlying etiology is unclear and malignancy cannot be ruled out, surgery should be performed promptly for both diagnosis and treatment.^1^ As in our case, an idiopathic rectosigmoid intussusception complicated with ischemia and bleeding was surgically removed to prevent obstruction, perforation, and persistent bleeding.

In conclusion, intussusception in adults is a rare entity with nonspecific presentations that may delay diagnosis and lead to life-threatening complications. Therefore, surgeons should be well-versed in various treatment options as definitive diagnosis may only be possible during laparotomy. CT remains an essential diagnostic modality, providing high accuracy and detailed visualizations. Treatment typically involves surgical resection of the affected segment of the bowel. Reduction may be attempted in case of a viable segment of the small intestine with no malignant features. However, in colonic intussusception, the significantly higher risk of malignancy requires a more cautious and definitive surgical approach.

## DISCLOSURES

Author contributions: K. Ali contributed to patient management, data collection, and manuscript preparation; S. Sohail was responsible for manuscript drafting and literature review; G. Dominguez Rivera supervised and provided critical revisions; V. Balderas approved the final manuscript. K. Ali is the article guarantor.

Financial disclosure: None to report.

Previous presentation: This case was presented at the ACG Annual Scientific Meeting, where it received the Outstanding Poster Presentation Award; October 25–30, 2024; Philadelphia, Pennsylvania.

Informed consent was obtained for this case report.
